# Burden and risk factors for *Schistosoma mansoni* infection among primary school children: A quantitative school-based cross-sectional survey in Busega district, Northern Tanzania

**DOI:** 10.1371/journal.pone.0280180

**Published:** 2023-01-12

**Authors:** George Ogweno, Vivian Mushi, Valeria Silvestri, Witness Bonaventura, Nyanda C. Justine, Mololo Noah, Furahini Yoram, Hussein Mohamed, Donath Tarimo

**Affiliations:** 1 Department of Parasitology and Medical Entomology, School of Public Health and Social Sciences, Muhimbili University of Health and Allied Sciences, Dar es Salaam, Tanzania; 2 National Institute for Medical Research, Mwanza, Tanzania; 3 Department of Zoology and Wildlife Conservation, College of Natural and Applied Sciences, University of Dar es Salaam, Dar es Salaam, Tanzania; 4 Maweni Regional Referral Hospital, Kigoma, Ministry of Health and Social Welfare, Dodoma, Tanzania; 5 Department of Clinical Oncology, School of Medicine, Muhimbili University of Health and Allied Sciences, Dar es Salaam, Tanzania; 6 Department of Environmental and Occupational Health, School of Public Health and Social Sciences, Muhimbili University of Health and Allied Sciences, Dar es Salaam, Tanzania; University of the District of Columbia, George Washington University School of Medicine and Health Sciences, UNITED STATES

## Abstract

**Background:**

Intestinal schistosomiasis is one of the most common neglected tropical diseases in Tanzania. Despite massive praziquantel administration, data from Northern Tanzania have reported a prevalence of up to 93.2%. Because the disease is focal, depending on host, environmental and intermediate host factors, there is a need to acquire data in specific settings to better tailor interventions. Therefore, the study assessed the prevalence and factors associated with persistent transmission of intestinal schistosomiasis among school-age children in Busega district, Northern Tanzania.

**Methods:**

A school-based cross-sectional study was conducted among 363 primary school children, randomly selected from school clusters in the Busega district. A single stool sample was collected from each child for *S*. *mansoni* ova and infection intensity examination using Kato-Katz. Factors related to intestinal schistosomiasis transmission were acquired through a questionnaire. A malacological survey was carried out to determine the *Biomphalaria* infectivity rate. Descriptive statistics and logistic regression analysis were conducted to analyse the association between *schistosoma* infection and factors related to transmission in this setting.

**Results:**

The prevalence of *S*. *mansoni* infection was 41.3% (95% CI: 36.3–46.5), statistically significantly higher among the younger group aged less than 11 years (46.4% vs 35.3%, p = 0.032). The intensity of infection was heavy in 1.6% of participants, moderate in 9.6%, and light in 30.9%. Studying at Mwamayombo Primary School (AOR = 2.50, 95% CI: 1.12–5.60) was the only factor significantly associated with *S*. *mansoni* infestations. The snail intermediate host belonged to *Biomphalaria sudanica* species, whose infectivity rate was quantified as 0.97%, thus confirming ongoing transmission in the area.

**Conclusions:**

There was a high prevalence of *S*. *mansoni* infection among school-age children in the Busega district. The presence of the infected *Biomphalaria sudanica* in the area documents the persistent transmission of the disease, favored by low knowledge and negative attitudes among school-aged children. Hence, the need for multi-approach intervention for schistosomiasis prevention and elimination.

## Introduction

*Schistosoma mansoni* (*S*. *mansoni*) is a trematode parasite acquired by contacting water bodies infested with its intermediate snail host of *Biomphalaria spps* shedding cercariae [1]. *S*. *mansoni* is the agent of hepato-splenic and intestinal schistosomiasis and a species of public health importance in the sub-Saharan setting. The prevalence of schistosomiasis and intensity of infection varies between geographical areas, age groups, and sex [2].

With the effort of intensive control and scaling up of programs by public and private partners, intestinal schistosomiasis remains a serious public health problem [3]. A key epidemiological feature of schistosomiasis is a focal distribution of the disease, with a highly variable prevalence and intensity of infection due to the different interactions of humans, intermediate host snails, and water presence in different settings [2]. Lack of access to water and sanitation and activities involving contact with infected water sources (domestic, recreational, or professional), put children, adolescents, and adults at risk of schistosome infection when exposed to contaminated water bodies [2]. Notwithstanding massive drug administration (MDA) programs put in place to prevent the disease, the prevalence of *S*. *mansoni* among school children is still high, and can reach up to 89.9% in some settings in African countries. However, it’s also true that MDA had a significant impact in reducing infection overall in Africa [4]. Due to environmental changes, *S*. *mansoni* has also been reported in a geographic area with no previous prevalence of human schistosomiasis [5–9].

The lack of knowledge related to schistosome agents, modality of infection, and preventive actions has been emphasized previously as a factor likely to influence the efficiency of preventive programs [10]. Negative attitudes have also been observed in addition to the lack of knowledge in sub-Saharan settings [11]. Heavy infections in children reduce physical and cognitive development, and when the infection is persistently not treated, it can lead to severe morbidity as well as mortality in serious cases [12]: anaemia, growth stunting, liver failure, portal hypertension, decreased quality of life, exercise intolerance, and infertility have been reported [13, 14]. Among African countries which are endemic to schistosomiasis and according to the available data on prevalence, Tanzania is ranked second after Nigeria [15]. *S*. *mansoni* is absent in the coastal area of Tanzania due to the absence of its intermediate snails host and thermal exclusion, but is dominant along the shores and islands of Lake Victoria, with the prevalence of intestinal schistosomiasis reaching 100% in previous surveys aimed at mapping the endemic areas in the country [16, 17]. Several studies conducted on the North-western region located along the Lake Victoria basin and the southern part of Lake Victoria showed the zone as highly endemic to *S*. *mansoni* infection with > 50% prevalence [16–21]. More than 53.2 million people in the country are at risk of the disease [22], and only school-aged children are reached by actual interventions in place [23].

In Tanzania, the use of mass praziquantel treatments as preventive chemotherapy was initiated in 2004–2005 as the main strategy for schistosomiasis prevention and control targeting school-aged children. According to the 2021 signed NTD master plan, with 184 councils targeted and a population of 51.5 million included, the geographical coverage of the programme reached 100% [23]. Notwithstanding these interventions, transmission continues in endemic settings throughout the country including the Busega district in the Simiyu Region. In 2017, a prevalence of 90.6% was reported among school-aged children in the district of Busega [17]. Because of the focal pattern of *S*. *mansoni* distribution [2], local data are crucial to guide interventions. Hence, the need to determine the current status burden and factors associated with the continuity of *S*. *mansoni* infection in this setting. This study investigated the magnitude and the factors for the continuation of *S*. *mansoni* transmission among primary school children in Busega district, Simiyu Region, Tanzania, to acquire information that could orient national health authorities to strategize and develop long-term interventions for schistosomiasis control and elimination, specifically targeting school aged children. Additionally, a malacological survey for the assessment of the presence of intermediate host and of the proportion of *Biomphalariae* shedding cercariae in this setting aimed at ascertaining factors for persistent transmission and informing programs through a multi-interventional perspective.

## Materials and methods

### Study setting

Busega district is bordered to the north by Lake Victoria and Bunda District, to the east by Bariadi District, and to the south by Magu District [24]. As of 2012, its population was 203,597 [17] and administratively, the district has thirteen wards. The district receives two rounds of rainfall per year, light rains around October to December and heavy rains around March to May, with an average annual rainfall ranging from 700 mm to 1000mm, and a mean temperature ranging between 18°C to 20°C during the rainy season and up to 32°C during the dry season. The rainy season favours the survival and breeding of *Biomphlaria* snails (intermediate hosts), thus the disease transmission [25]. Schistosomiasis is among the top ten causes of morbidity and mortality in this setting [17].

The district’s major economic activities are farming and fishing which could predispose the community members at risk of acquiring intestinal schistosomiasis. Schistosomiasis infection transmission is facilitated by the presence of fresh water bodies [17]. Water coverage in the district was 39.5% of the total population of 234,732 in 2018. Busega District Council has four types of water sources used in the community which include lakes, boreholes, shallow wells, and rainwater [26]. The Busega district was chosen as the study site because of the documented history of a high intestinal schistosomiasis prevalence [17].

### Study design

A school-based cross-sectional study was conducted to establish the prevalence, intensity, and factors associated with the continuity of *S*. *mansoni* transmission among primary school children in Busega, Simiyu.

### Study participants’ inclusion and exclusion criteria

The study participants were primary school children living along the shore of Lake Victoria in the Busega district. The inclusion criteria were being primary school children in one of the two schools of the selected ward of the Busega district, having completed the structured questionnaire and provided a stool sample. Parental consent was also needed for eligibility. Having received praziquantel in the previous six months was among the criteria for exclusion. Also, participants that failed to answer the questionnaire or provide stool samples were excluded from the study.

### Sampling and sample size

**Fig 1:**
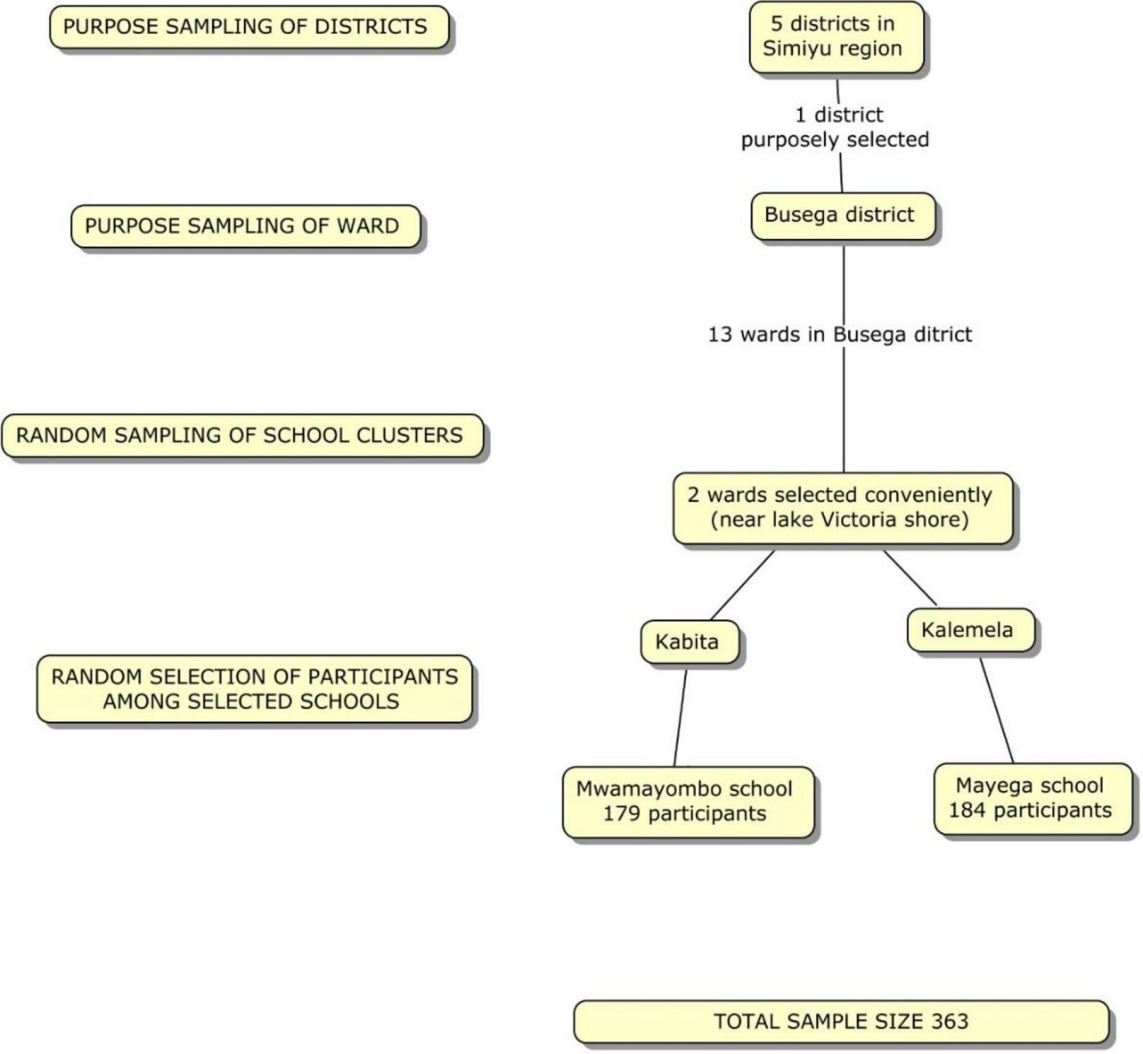
Flow chart of sampling technique.

The study sample size was estimated using the following formula [27] n = Z^2^p [1-p]/ɛ ^2^ whereby; n = the minimum estimated sample size; Z = standard normal deviate (1.96 for 95% confidence interval); p = expected proportion, (0.385) from previous studies in Lake Victoria region [22], ɛ is the margin of error, settled at 5%; a correction for design effect of 2 and for 5% of anticipated non-response rate was also applied. According to sample size calculation, 364 participants were enrolled by multi-stage cluster sampling technique as described in detail in Fig 1.

### Tools and methods for data collection

#### Questionnaire survey

A three-section structured questionnaire written and administered by the interviewer in the kiswahili language was used to acquire information on demographic characteristics, knowledge, and attitudes on intestinal schistosomiasis and water contact practices among primary school age children (S1 and S2 Files).

#### Observation checklist

In measuring the water contact practices, an observation checklist and questionnaire were used, which specified the type of activities performed, the frequency and time of water contact, and their association with *S*. *mansoni* infection (S3 File).

#### Stool samples collection and laboratory investigation

One stool sample was collected from each participant in pre-labelled, small, clean, dried, and leak proof stool containers and then transported to the National Institute of Medical Research (NIMR) Parasitology laboratory in Mwanza for examination. Collected stool specimens were examined by experienced medical laboratory technicians using Kato- Katz egg counting technique as documented in the host laboratory standard operation procedure [28]. Specimens that were found to have *S*. *mansoni* eggs were recorded as infected, and the number of eggs in each specimen was counted; the intensity of infection was determined by multiplying the total number of eggs per slide by a factor of 24 and recorded as the geometric mean, expressed as number of eggs per gram of faeces (EPG) [28].

### Malacological section

#### Inclusion and exclusion criteria for malacological survey

The intermediate snail’s host population of the *Biomphalaria species* were sampled in water bodies in the study area. Dead snails were excluded from the data analysis. All *Biomphalaria* snails present in the frequently contacted water bodies present in the study area were eligible for the study.

#### Sampling and sample size for snails

The total sample size for collected snails depended on the availability and quantity of snails at each collection site, localized with the Global Positioning System (GPS), in which populations appeared to be in contact with fresh water.

### Snail collection and shedding

Snail sampling sites were selected along the shorelines where children have direct water contact. The sanitary inspection checklist was used to register water pollution activities and snail flourishing environment, as recorded in S3 File. At every site, the snails were collected by an experienced malacologist by scooping in an area of 5m^2^ and lengths of 10 meters along streams and the lake Shoreline. The collection of snails lasted 30 minutes at each site per World Health Organization (WHO) guidelines [29]. Snails were collected with forceps and stored in labelled containers filled with water and lined by grass cover from the same water contact points and transported to the NIMR Parasitology laboratory.

**Fig 2:**
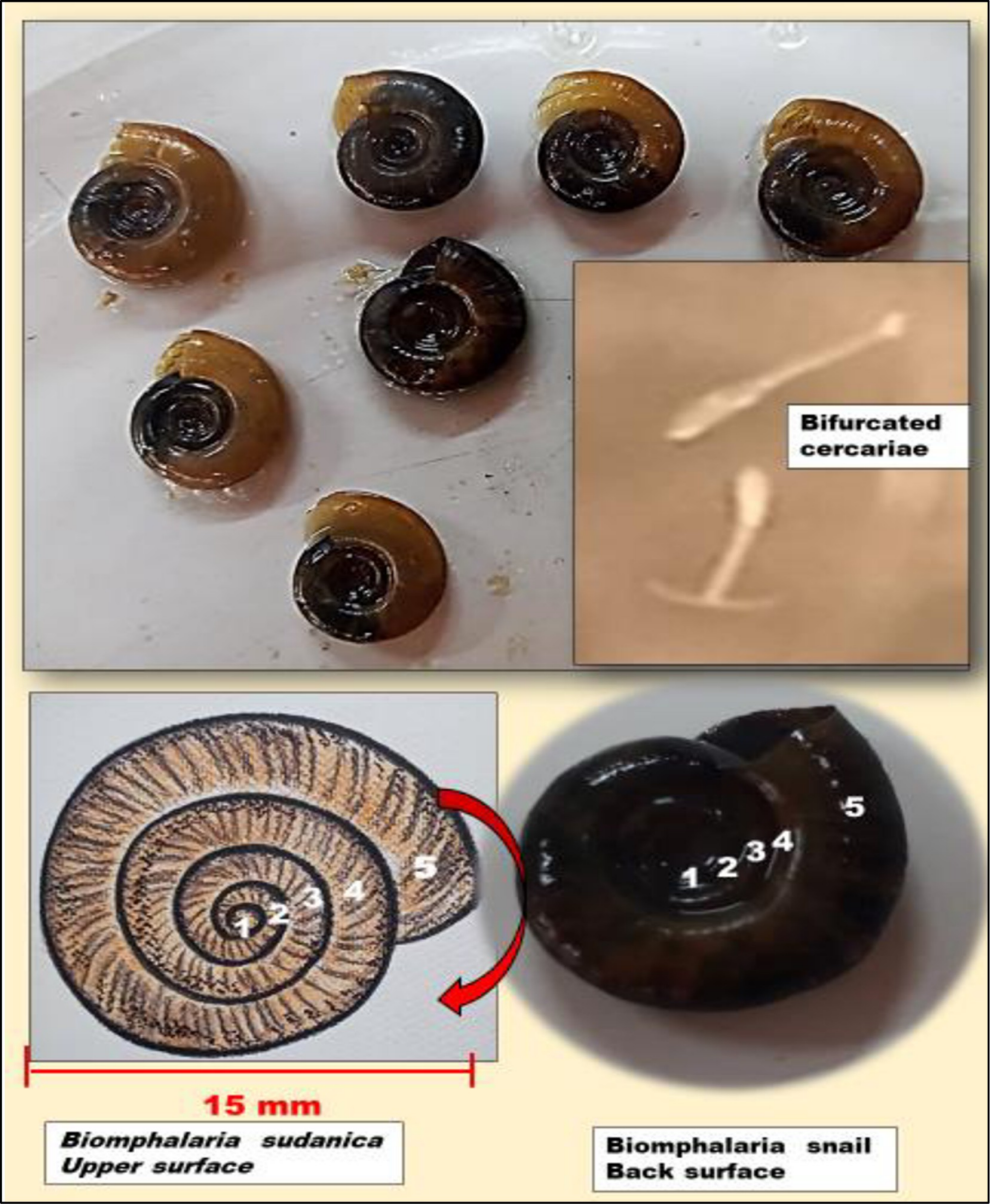
Sampled snails of *Biomphalaria sudanica* species. Notice the number of shell coils (5) which differentiate *B*. *sudanica* from other species. *Biomphalaria* is right coiled (clockwise coil). Shedded bifurcated cercariae are also visible in the picture. The bifurcated cercariae indicate human *schistosoma* cercariae.

All collected snails were morphologically identified using the field guide [30] based on shell morphological characteristics using standard keys developed by Kristen [30]. Morphological details of the collected snails have been provided in Fig 2. The snails were then placed individually in 24-well culture plates containing 1ml of clear filtered water from the same source as the site of the collection spot and exposed for one hour to indirect sunlight to induce cercaria shedding at the early peak shedding time of midday.

The wells of the plate were then examined for the presence of bifurcated cercariae (of mammalian origin) under a dissecting microscope. Snails that were observed not to shed cercariae on the first exposure were re-exposed on the second day for cercariae shedding. Cercarial shedding was calculated and reported in percentage as the number of *Biomphalaria* snails that tested positive over the total number of sampled snails [31]. The results were recorded in snail collection and report form (S4 File).

### Quality control

The questionnaire was pre-tested in Nyamadoke primary school at Ilemela district in Mwanza, among 36 students, corresponding to 10% of the sample size required for the study.

### Statistical analysis

The primary data collected were cleared before coding, double entered to minimize errors, and analysed with the statistical software STATA version 14 (STATACorp Inc., TX, USA). The main outcome variables in this study were schistosomiasis infection according to the presence or absence of *S*. *mansoni* eggs in stool samples on microscopic assessment. Descriptive statistics were used to obtain frequency tables, proportions, and their 95% CI. The association between intestinal schistosomiasis knowledge, attitudes, and water contact practices with socio-demographic characteristics was assessed by the Chi-square test or Fishers exact where necessary at a significance level of 0.05.

Knowledge was measured using seven multiple selection questions carrying a weight of 6–12 marks per question. The scores for all options were added, ranging from 59–85, and then divided into three categories; 59–68 was classified as a low level of knowledge, 69–78 as moderate, and ≥ 79 as high. On the attitude assessment, the individual scores were added to obtain the total attitude score ranging from 12–60. The mean attitude score (45), calculated from the total scores, aided in the classification of attitude as positive (≥ 45 scores) or negative (12–44 scores) attitude.

As for the analysis of malacological results, the number of snails collected per 30 min at a constant collection rate per site was reported as high abundant (> 30 snails), moderate abundant (10–30 snails), and low abundant (< 10 snails). To identify the variables with p < 0.25 to include in the multivariate logistic regression, a univariate logistic regression analysis was performed. The dependent variable in the logistic regression analysis was the actual laboratory positivity for *S*. *mansoni*. The results were reported as COR and AOR with a 95% CI.

### Ethical considerations

Ethical approval to conduct this study was sought from the Institutional Review Board of the Muhimbili University of Health and Allied Sciences (MUHAS) with a number of protocol REC 03-2022-1049. Permission was acquired from the local authorities, respectively the Regional Administration Secretary (RAS) and Regional Medical Officer (RMO) Simiyu Region. Written informed consent was acquired from teachers on behalf of the parents who verbally consented for their children to participate. Information was kept confidential. Treatment was planned for the participants who were found to be positive on laboratory investigation.

## Results

### Socio-demographic characteristics of the study participants

A total of 363 primary school children of both sexes (182 male and 181 female) and with a mean age of 10.3 ± 2.5 years (ranging from 6 to16 years) were interviewed. The response rate was 99.73%. Both schools were equally represented, with 50.7% participants from Mayega and 49.3% from Mwamayombo (Table 1).

**Table 1:** Socio-demographic factors, the prevalence of *S*. *mansoni* infection, levels of knowledge, and attitudes among the participants.

Variables	Number of participants n (%)	*S. mansoni* prevalence	p-value	Number of participants	Knowledge			p-value	Attitudes		p-value
					Low n (%)	Moderate n (%)	High n (%)		Positive n (%)	Negative n (%)	
**Sex**	** **	** **	** **	** **	** **	** **	** **	** **	** **	** **	** **
Male	182 (50.1)	78 (42.9)	0.552	129 (49.0)	09 (7.0)	60 (46.5)	60 (46.5)	0.995	53 (39.6)	81 (60.4)	0.525
Female	181 (49.9)	72 (39.8)		134 (51.0)	09 (6.7)	62(6.7)	63 (47.0)		56 (43.4)	73 (56.6)	
**Age group**											
<11 Years	196 (54)	91 (46.4)	0.032*	118 (44.9)	04 (3.4)	49 (41.5)	65 (55.1)	0.018*	50 (42.4)	68 (57.6)	0.783
≥11Years	167(46)	59 (35.3)		145 (55.1)	14 (9.7)	73 (50.3)	58 (40.0)		59 (40.7)	86 (59.3)	
**Class level**											
Classes 1–4	203 (55.9)	92 (45.3)	0.081	124 (47.1)	04 (3.2)	53 (42.7)	67 (54.0)	0.020*	52 (41.9)	72 (51.8)	0.879
Classes 5–7	160 (44.1)	58 (36.2)		139 (52.9)	14 (10.1)	69 (49.6)	56 (40.3)		57 (41.0)	82 (59.0)	
**School**											
Mayega	184 (50.7)	55 (29.9)	0.001*	143 (54.4)	15(10.5)	63 (44.1)	65 (45.4)	0.036*	67 (46.9)	76 (53.1)	0.034*
Mwamayombo	179 (49.3)	95 (53.1)		120 (45.6)	03 (2.5)	59 (49.2)	58 (48.3)		42 (35.0)	78 (65.0)	

*Statistically significant (p< 0.05)

### Prevalence and intensity of *Schistosoma mansoni* infection among the participants

The overall prevalence of *S*. *mansoni* infection was 41.3% (95% CI: 36.3–46.5), with no statistically significant difference in prevalence according to gender (42.9% vs 39.8%; p = 0.552). Prevalence was significantly higher among the younger group aged less than 11 years rather than the older (46.4% vs 35.3%, p = 0.032), and in Mwamayombo primary school when compared to Mayega primary school (53.1% vs 29.9%, p<0.001) (Table 1). The majority of participants had a light intensity of *S*. *mansoni* infection (30.9%), followed by a moderate intensity of 9.6% and heavy intensity of 1.6%.

### Knowledge on schistosomiasis infection, transmission, and prevention among the participants

More than two-thirds (72.4%) of primary school children had heard about schistosomiasis, in the majority of cases in school (83.3%). Less than two-thirds (63.5%) acknowledged intestinal schistosomiasis (*S*. *mansoni*) as a type of schistosomiasis and more than three quarters (79.1%) correctly reported bathing in the lake/river as a way of transmission. Avoiding swimming/bathing in water bodies was the most frequently mentioned prevention method (87.8%) (Table 2).

**Table 2:** Summary of knowledge on intestinal schistosomiasis among the participants.

Variable	n (%)	95% CI
**Heard about schistosomiasis**	263 (72.4)	68.3–76.6
**Sources of information**		
School	219 (83.3)	78.1–87.7
Radio	104 (39.5)	33.6–46.0
Television	39 (14.8)	10.2–19.0
Health facility	71(27.0)	21.9–33.3
Family/Friend/Neighbor	137 (52.1)	46.6–57.8
Health Program	22 (8.4)	5.4–12.0
Other sources	02 (0.8)	0.0–2.1
**Types of schistosomiasis**		
Intestinal schistosomiasis (*S*. *mansoni*)	167 (63.5)	58.5–69.4
Urinary schistosomiasis (*S*. *haematobium*)	151 (57.4)	51.4–63.1
Others	03 (1.1)	0.0–2.6
Don’t know	47 (17.9)	12.9–22.6
**Schistosomiasis transmission methods**		
Walking in the infected lake/river water	135 (51.3)	44.9–57.5
Bathing in the lake/river	208 (79.1)	73.9–84.0
Swimming in the lake/river	173 (65.8)	60.4–71.8
Drinking dirty water	131 (49.8)	44.1–56.0
Eating contaminated food	37 (14.1)	10.3–18.7
Stepping onto the feces/urine of the infected person	68 (25.9)	21.0–31.3
Working out in the rain	08 (3.0)	1.2–5.2
Sexual contacts	11 (4.2)	2.2–6.6
Drinking unboiled water	50 (19.0)	14.2–24.1
Others	01 (0.4)	0.0–1.2
**Sign and symptoms of schistosomiasis**		
Abdominal pain/colic	219 (83.6)	79.0–87.8
Diarrhea/loose stool	79 (30.2)	24.1–35.5
Blood in stool	125 (47.7)	41.6–53.8
Vomiting blood	47 (17.9)	14.1–22.9
Swollen abdomen/ascites	34 (13.0)	9.2–16.8
Flatulence/dyspepsia	31 (11.8)	7.6–16.0
Body weakness/fatigue	48 (18.3)	13.4–23.3
**Do snails transmit schistosomiasis**		
Yes	124 (47.1)	40.3–53.6
No	78 (29.7)	23.6–350
Don’t know	61 (23.2)	17.9–28.5
**Ways to prevent schistosomiasis**		
Taking preventive treatment	123 (46.8)	40.7–53.0
Avoid swimming/bathing in water bodies	231 (87.0)	84.0–91.2
Wearing protective clothing while in contact with water	73 (27.8)	22.4–33.1
Leaving water to settle for almost 8 hours before using it	42 (16.0)	12.2–20.4
Boiling water or leaving it out in the sun before drinking it	75 (28.5)	22.6–33.7
Others	03 (1.1)	0.0–2.4

Out of 263 participants who heard about schistosomiasis, about (46.8%) had a high level of knowledge about *S*. *mansoni* infection, 46.4% had a moderate level of knowledge, and 6.8% had a low level. A statistically significant lower level of knowledge was observed among participants of ≥ 11 years (p = 0.018), classes 5–7 (p = 0.020), and Mayega primary school (p = 0.037) (Table 1).

### Attitudes towards intestinal schistosomiasis control strategies among the participants

**Fig 3:**
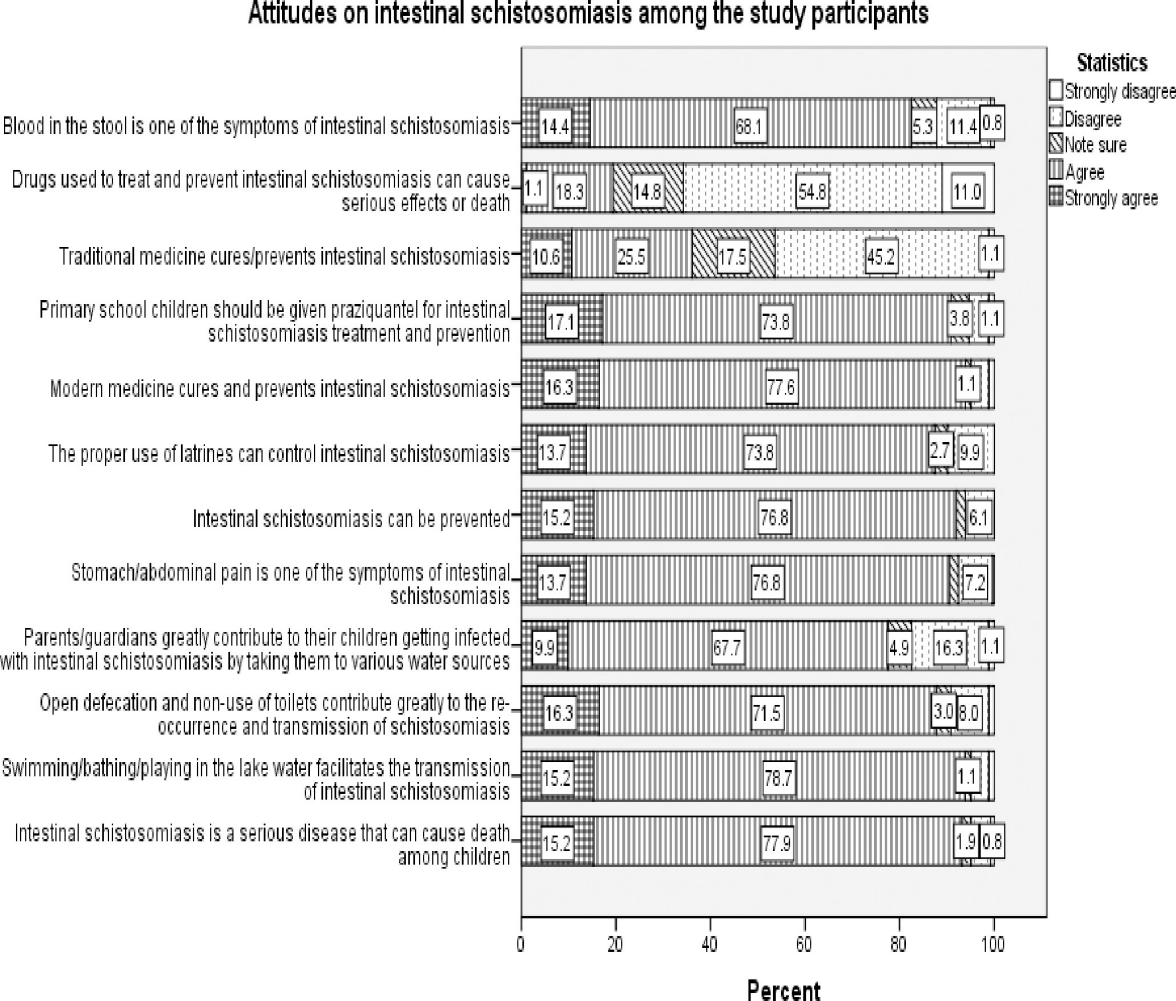
Attitudes on intestinal schistosomiasis among the study participants.

Out of 263 participants, 55.9% had positive attitudes toward intestinal schistosomiasis control strategies while 44.1% had negative attitudes (Table 1). More than three quarters (79.1%) agreed that schistosomiasis is transmitted through swimming in the lake, more than two thirds recognized open defecation as a risk factor for transmission (71.5%) and 67.7% acknowledged taking children to various water sources as a risk factor for infection. More than two thirds 73.8% correctly mentioned the proper use of toilets for control of *S*. *mansoni* transmission and 75.3% agreed on modern medicine and the use of praziquantel to control schistosomiasis (Fig 3).

### Association between knowledge and attitudes with the burden of *S*. *mansoni* infection

Less than half of the primary school children (41.0%) with a moderate level of knowledge were positive for intestinal schistosomiasis. Less than half of the primary school children (44.0%) with negative attitudes were positive for intestinal schistosomiasis. However, the observed difference was not statistically significant, indicating no association between schistosomiasis knowledge and infection (Table 3).

**Table 3:** Association between knowledge and attitudes with the burden of *S*. *mansoni* infection.

Variable	Total	Schistosomiasis prevalence n (%)	p-value
**Level of knowledge**	** **	** **	** **
Low	18	06 (33.3)	0.822
Moderate	122	50 (41.0)	
High	123	50 (40.6)	
**Attitudes towards schistosomiasis**			
Positive	147	55 (37.4)	0.282
Negative	116	51 (44.0)	

### Water practices and burden of *S*. *mansoni* among the participants

Among the water contact practices analyzed, only going routinely with parents to the water sources was a significant factor for infection (p = 0.005). Out of 66.7% of children routinely going with parents to the water sources, 33.9% were found to be positive for *S*. *mansoni* (Table 4).

**Table 4:** Water contact practices of the study participants.

Water contact practices	Total n (%)	Schistosomiasis test with positive results n (%)	p-value
**Going to the lake**	252 (95.8)	101 (40.1)	0.722
**Reason for visiting the lake**			
Bathing	225 (89.3)	91 (40.4)	0.733
Fishing	10 (4.0)	02 (20.0)	0.186
Fetching water	215 (85.3)	88 (40.9)	0.506
Washing clothes/utensils	174 (69.1)	63 (36.2)	0.061
Playing/recreation	39 (15.5)	17 (43.6)	0.627
Swimming	165 (65.5)	67 (40.6)	0.814
Transport /fording in transit in the lake water	06 (2.4)	01 (16.7)	0.236
Do you normally go with your parent to water sources	168 (66.7)	57 (33.9)	0.005*
**Time spends in the lake**			
Very Short time <5 Minutes	21 (8.3)	06 (28.6)	0.122
Short time 5–15 Minutes	128 (50.8)	54 (42.2)	
Long time 15–60 Minutes	89 (35.3)	39 (43.8)	
Very Long time > 1hour	14 (5.6)	02 (14.3)	
**Time of day you go to the lake**			
Morning	105 (41.7)	40 (38.1)	0.133
Midday	44 (17.4)	13 (29.6)	
Afternoon	103 (40.9)	48 (46.6)	

*Statistically significant (p< 0.05)

### Risk factors associated with *S*. *mansoni* infection among the participants

In a multivariate analysis of the factors associated with *S*. *mansoni* infection, only the fact of going to school in Mwamayombo was significantly associated with infection, with an AOR of 2.50, 95% CI: 1.12–5.60 (Table 5).

**Table 5:** Risk factors associated with *S*. *mansoni* infection among the participants.

Variable name	Crude Odds Ratio			Adjusted Odds Ratio		
	COR	95% CI	p-value	AOR	95% CI	p-value
**Age group**						
< 11 years	Ref					
≥11 years	0.63	0.41–0.96	0.033	0.86	0.47–1.57	0.632
**School**						
Mayega	Ref					
Mwamayombo	2.65	1.72–4.08	0.001	2.5	1.12–5.60	0.025*
**Awareness of schistosomiasis for family/Friend**						
Yes	Ref					
No	1.92	1.16–3.16	0.011	1.04	0.57–1.90	0.88
**Playing in the dirty water**						
Yes	Ref					
No	1.72	1.05–2.84	0.032	1.37	0.77–2.42	0.273
**Snails in water bodies transmit schistosomiasis**						
Yes	Ref					
No	2.06	1.15–3.67	0.015	0.97	0.45–2.08	0.941
Don’t know	1.27	0.67–2.38	0.465	0.98	0.47–2.01	0.959
**Do you go with your parents to the lake**						
Yes	Ref					
No	2.14	1.25–3.65	0.005	1.12	0.53–2.37	0.76
**Water conduct practice-washing clothes**						
Yes	Ref					
No	1.67	0.97–2.87	0.062	1.11	0.60–2.06	0.731
**Time spent in the lake**						
Very short time	Ref					
Short time 5–15 Minutes	1.82	0.66–5.01	0.243	1.1	0.36–3.31	0.86
Long time 15–60 Minutes	1.95	0.69–5.49	0.206	0.99	0.31–3.19	0.95
Very Long time > 1 Hour	0.42	0.07–2.45	0.333	0.34	0.05–2.23	0.263
**Time of day normally go to the lake**						
Morning	Ref					
Midday	0.68	0.32–1.45	0.321	1.11	0.45–2.69	0.826
Afternoon	1.41	0.82–2.46	0.215	1.64	0.78–3.42	0.191

*Statistically significant (p< 0.05)

### Prevalence of cercaria shedding among the collected *Biomphalaria* snails

A total of 9 sites were visited along the Lake Victoria shoreline around Mayega and Nyakaboja villages. All 9 sites were heavily polluted with human faeces (open defecation). No public toilets were available on the shores of the lake. Out of 9 sites, 7 sites were classified as highly abundant (> 30 snails) while the remaining 2 sites (< 10 snails) were classified as low abundant in snails. A total of 518 *Biomphalaria* snails were collected, all classified as *Biomphalaria sudanica*. Five (0.97%) snails collected during the survey were found to be shedding human schistosome cercariae (Table 6).

**Table 6:** Prevalence of *Biomphalaria sudanica* cercariae shedding per site.

Village	Site	Total number of snails collected	Number of snails infected (%)
Mayega	Ndalawa	03 (0.6)	0 (0.0)
	Safari	35 (6.8)	0 (0.0)
	Solwe	133 (25.7)	1 (0.75)
	Yebote	07 (1.4)	0 (0.0)
Nyakaboja			
	Bukabile 1	88 (17.0)	1 (1.14)
	Bukabile 2	33 (6.4)	0 (0.0)
	Bukabile 3	56 (10.8)	1 (1.79)
	Mwakabote	94 (18.1)	2 (2.13)
	Mwembeni	69 (13.3)	0 (0.0)
**Total**	** **	**518**	**5 (0.97)**

## Discussion

The study established the burden of *S*. *mansoni* infection and the factors associated with continuity of transmission among primary school children in the Busega district, Simiyu region, Tanzania. Despite the ongoing school-based MDA for more than a decade and health education programs in schools, the overall prevalence of *S*. *mansoni* was 41.3%, which is still a high prevalence. This could be explained by three factors. First, despite the frequency of MDA distribution being regular, issues of compliance could undermine the efficacy of the programs in place and need to be further studied and eventually addressed. Additionally, previous studies have observed a poor efficacy of praziquantel in the immature stage of the parasite, which, after 4–6 weeks, can grow and start laying eggs, contaminating the environment, and favouring regression to pre-treatment prevalence [17]. Second, we have confirmed through a malacological survey the supportiveness of the environment towards the snail intermediate hosts. Last, we have reported frequent water contact activities that could expose to infection risk, even though no specific water practice was significantly associated with the infection. However, our observed prevalence is lower if compared to the study conducted in the Kabita ward located in the same district in 2015, which was reported to be up to 90.6%. Authors have reported that the high prevalence observed in that setting could have been the result of the interruption of MDA administration in the area since 2016 [17]. In our setting, the administration of MDA was regularly implemented.

When considering data from other countries, a prevalence similar to our findings was recently reported in western Kenya (41.3%) [32]. Our data also agree with the prevalence of *S*. *mansoni* observed in Ituri Province, in the north-eastern Democratic Republic of the Congo, which ranged between 52.8 to 95.0% in 2017 [33]. On the contrary, in Ethiopia, the observed prevalence was 11.2% among pre-school aged children, which is lower compared to the findings of the current study and highly variable among different sites in Ethiopia. This difference can be explained by a variety of water sources, time of the survey, environmental and socio-economic factors [34], summarizing, the fact that intestinal schistosomiasis is a focal disease in which many factors contribute to the observed prevalence [2]. The role of WASH in the transmission of intestinal schistosomiasis hasn’t been described exhaustively by previous studies [35]. One of the schools in the study area lacked a clean water supply system, exposing children to infection when collecting water from the lake for toilet use at school. Additionally, in another school that had a clean water supply in place, the experiences of water shortages could also induce pupils to fetch water from the lake for school sanitation.

In terms of the observed prevalence of *S*. *mansoni* infection (42.9% in males vs. 39.8% in females; p = 0.552), there was no statistically significant gender difference. Similar to another study analyzing prevalence among school-aged children in North-East Tanzania with no gender difference reported [17]. We have observed a higher prevalence of *S*. *mansoni* infection among participants younger than 11 years when compared to the older group (46.4% vs 35.3%). Previous studies have observed a higher prevalence of *S*. *mansoni* in pre-adolescents than adolescents, likely due to the low immunity at a younger age which may contribute to an increased susceptibility to infection [17]. Mwamayombo primary school reported a 53.1% prevalence of *S*. *mansoni* infection, which was significantly higher when compared to the other schools. A high prevalence could be favoured in this setting because even though there are flush toilets, water is not available at school, and water is collected by children at the lake.

Based on the Kato Katz slide reading results, 1.6% of the participants had a higher intensity of *S*. *mansoni* infection. This was lower compared to the study conducted in the Nyasa district, where the prevalence of *S*. *mansoni* was 15.1%, with 17.8% of participants having a heavy infection [36]. Variations in study settings and the interventions carried out previously could explain these differences.

The majority of the study participants had heard about schistosomiasis, but still, 6.8% of them only had a low level of knowledge about the disease. A low level of knowledge has been denounced in previous studies. Data from Ijinga Island in Lake Victoria showed that only 17% of the assessed school-age participants had relevant related knowledge, more frequently those in high grades (grades 6–7), or who acquired knowledge at school or had a previous schistosoma diagnosis [37]. The role of knowledge on the cause of schistosomiasis, risk factors, and mode of transmission in contributing to behavioural practices and participation in control interventions has been previously emphasized [10]. Interestingly, even among those participants who heard about schistosomiasis, 55.9% had a negative attitude about the disease. Negative practices could likely be favoured by insufficient sanitation measures and safe water supply, which would make feasible healthy habits, impacting negatively on the effectiveness of preventive interventions [10].

In our study, most of the participants reported going to the lake. Bathing, fetching water, washing clothes, and swimming were among the most frequently reported activities, but none of the water-contact practices was significantly associated with schistosoma infection. Previous studies have observed increased water contact through swimming, fishing, washing clothes, and also during irrigation practices in children living in lake areas [36]. The lack of latrine available close to the lakes increased the *S*. *mansoni* infection as result of poorly handled faecal material in Tanzania [17], and living in proximity to the water sources has been reported as a contributing factor to the transmission of *S*. *mansoni* infection [19]. On the other hand, access to safe and clean drinking water reduced *S*. *mansoni* infection, as reported on the prevalence and correlates of intestinal schistosomiasis infection among school-aged children in North-Western Tanzania [17]. Access to safe water has been considered one of the major control strategies of *S*. *mansoni* infection [35].

The global geographic distribution of *S*. *mansoni* is influenced by the presence of susceptible species of *Biomphalaria* freshwater snails that support the parasite’s transformation into infective stages in favourable environments. *Biomphalaria species* have shown strong dispersal capacities. Cercarial densities are directly associated with intestinal schistosomiasis infection in humans [37]. We observed that Lake Victoria’s shoreline in Busega district, where people had direct water contact in the lake water, is a hotspot breeding site for *Biomphalaria* snails. Five out of 518 collected *Biomphalaria* snails shedded human schistosoma cercariae, for an observed prevalence of shedding of 0.97%, confirming the presence of *S*. *mansoni* in the environment, as also reported by a malacological study done in Chad. In this setting, *Biomphalaria spp* was also detected, which showed with a prevalence of shedding *S*. *mansoni* of 0.93% [38]. Also, a previous study in Gombe’s ecosystem in western Tanzania reported the presence of *Biomphalaria pfeiffer* with a prevalence of 12% cercarial shedding [39]. Compared to this study, the prevalence of shedding that we have observed is lower but could be biased by the smaller sample size.

## Study limitations

There are some limitations to this study to be acknowledged. First, the collection of data related to knowledge, attitudes, and practices was done through a questionnaire containing questions prone to recall bias. One additional limit is due to the collection and processing of only one stool sample hence the possibility of underestimation of schistosoma prevalence. Finally, the collection of snails was carried out only once, thus potentially biasing the result of the malacological survey and cercaria shedding prevalence.

## Conclusions and recommendations

Our study has observed a high prevalence of *S*. *mansoni* infection among primary school children in the Busega district in the Simiyu region, Tanzania, with the majority of infections being of light and moderate intensity. Studying at Mwamayombo Primary School was the only factor significantly associated with positivity of stool samples for the presence *Schistosoma mansoni* eggs. The malacological survey confirmed the presence of the infected snails at the sites along the lakeshore, with a low cercaria shedding among *Biomphalaria* snails, allowing persistent transmission in areas with high human contact with fresh water.

Our findings suggest that an integrated approach including malacological interventions and sanitation intervention, that could address those factors that are likely to impair the efficiency of the ongoing programs, is needed to achieve a significant reduction of intestinal schistosomiasis in endemic areas, which has not been achieved with MDA alone. Health education through meetings at the community level should be strengthened to address the knowledge gap, especially in lower level classes and among younger students.

## Supporting information

S1 File This is the questionnaire in Kiswahili. (DOCX)

S2 File This is the questionnaire in English. (DOCX)

S3 File This is the observation checklist. (DOCX)

S4 File This is the snail collection and report form. (DOCX)

S1 Data The data set used for analysis. (XLS)
