# Supplementary material for: Burden and risk factors for Schistosoma mansoni infection among primary school children: A quantitative school-based cross-sectional survey in Busega district, Northern Tanzania
Source: PLoS One. 2023 Jan 12;18(1):e0280180. doi: 10.1371/journal.pone.0280180 (PMC9836289; doi:10.1371/journal.pone.0280180)
Supplement: S1 File — (DOCX) [file pone.0280180.s001.docx]

# S1 File-Kiswahili questionnaire

**KIWANGO CHA MAAMBUKIZI YA KICHOCHO CHA TUMBO MIONGONI MWA WATOTO WA SHULE ZA MSINGI: UFAHAMU NA MITAZAMO YAO KATIKA WILAYA YA BUSEGA, SIMIYU TANZANIA**

**Utangulizi**

Habari. Naitwa--------------------------------- ­­­­­­­­­­­­­­­­­­­­­­­­­­kutoka Chuo kikuu cha sayansi ya afya na tiba Muhimbili (MUHAS). Ninafanya utafiti kuhusiana na Kichocho cha tumbo kwa Watoto wa shule za msingi katika jamii yenu. Ningependa kujifunza kutokana na uzoefu wako ni namna gani Kichocho cha tumbo kinaambukizwa. Ninafuraha kua umekubali kuzungumza namimi. Taarifa zote zitakazo kusanywa kutoka kwako zitatumika pasipo kutaja jina lako. Mahojiano haya yatachukua takribani dakika ishirini na thelathini.

Una swali lolote au maoni kabla hatujaanza?

**Mhojaji:** Endapo kuna swali lolote tafadhali jaribu kujibu kabla ya kuanza mahojiano

Naomba nikuulize maswali machache kuhusiana na historia yako.

| **Namba ya utambulisho ya dodoso:** |  |
| --- | --- |
| **Code namba za mhojaji** |  |

**A: Utambulisho na taarifa za mshiriki**

| **No.** | **Swali na mchujo** | **Namba kwa kundi** |
| --- | --- | --- |
| 1. 1 | Jinsia ya mshiriki | 0 Ke  1 Me |
| 1. 2 | Una umri gani? (Katika miaka iliyo kamilika) |  |
| 1. 3 | Unasoma Darasa la ngapi? |  |

**B: Uelewa na ufahamu juu ya Kichocho**

| **No.** | **Swali na mchujo** | **Coding categories** |
| --- | --- | --- |
| 1. 8 | Umewahi kusikia ugonjwa wa Kichocho? | 1 Ndiyo **→ Endelea 5**  0 Hapana **→ Maliza dodoso** |
| 1. 9 | Ulisikia wapi? (Wapi ulipata taarifa kuhusu Kichocho)?  **(Zungushia jibu zaidi ya moja)** | 1. Shuleni 2. Redioni 3. Runinga 4. Kituo cha kutolea tiba (kituo cha afya) 5. Familia /Marafiki/Jirani 6. Kampeni za afya 7. Nyingine   Fafanua………………………………………………………… |
| 1. 10 | Taja aina za Kichocho unazozi fahamu  **(Zungushia jibu zaidi ya moja)** | 1. Kichocho cha tumbo (S. mansoni) 2. Kichocho cha mkojo (S. haematobium) 3. Nyingine   Fafanua……………………………………………………...  88 Sijui |
| 1. 11 | Ni kwa namna gani watu wanaambukizwa kichocho?  **(Zungushia jibu zaidi ya moja)** | 1. Kutembea ndani ya maji (Kugusa maji) Ziwa/Mto wenye vimelea 2. Kuoga ndani ya ziwa/mto wenye vimelea 3. Kuogelea ndani ya Ziwa/Mto wenye vimelea 4. Kunywa maji machafu 5. Kula chakula kichafu 6. Kukanyaga kinyesi/mkojo wa mtu mwenye maambukizi 7. Kufanya kazi kwenye mvua 8. Kwa njia ya kujamiiana 9. Kunywa maji yasiyo chemshwa 10. Nyingine   Fafanua …………………………………………………….  88 Sijui |
| 1. 14 | Ni dalili zipi anapata mtoto mwenye maambukizi ya kichocho cha tumbo?  **(Zungushia jibu zaidi ya moja)** | 1. Maumivu ya tumbo 2. Kuhara/choo chepesi 3. Damu katika kinyesi 4. Kuhara damu 5. Kutapika damu 6. Kujaa/kuvimba tumbo 7. Kukojoa damu 8. Maumivu wakati wa kukojoa 9. Kuvimba sehemu ya chini ya miguu na sehemu nyingine za mwili 10. Kupungukiwa damu 11. Mvurugiko wa tumbo 12. Mwili kuwa dhaifu/kuchoka mwili 13. Nyingine   Fafanua…………………………………………………………  88 Sijui |
| 1. 16 | Ni zipi tabia au shughuli zinazo peleka watu kupata maambukizi ya Kichocho cha tumbo?  **(Zungushia jibu zaidi ya moja)** | 1. Kujisaidia haja kubwa hovyo (e.g., porini au karibu na vyanzo vya maji) 2. Kuoga ziwani/mtoni 3. Kuchezea maji machafu 4. Kuchezea udongo 5. Kula vitu vichafu 6. Kutembea bila viatu 7. Nyingine (fafanua)…………………............................ |
| 1. 18 | Ni kwa na namna gani kichocho huenea kwenye vyanzo vya maji? (Ziwa, Mto, Bwawa, n.k)?  **(Zungushia jibu zaidi ya moja)** | 1. Kukojoa ndani ya vyanzo vya maji 2. Kujisaidia ndani ya vyanzo vya maji 3. Kumwaga chakula kilicho lala/kiporo ndani ya vyanzo vya maji 4. Kurogwa/ushirikina 5. Kufanya mapenzi 6. Nyingine(Fafanua)………………………………………………….   88 Sijui |
|  | Unafikiri konokono ndani yam aji wanaweza kusababisha kuenea kwa kichocho | 1. Ndiyo 2. Hapana   88 Sijui |
| 1. 34 | Ni kwa namna gani unajiepusha kupata maambukizi ya kichocho cha tumbo?  **(Zungushia jibu zaidi ya moja)** | 1. Kutumia kinga tiba m.f. MDA 2. Kuepuka kuogelea/kuoga kwenye vyanzo vya maji m.f ziwa, mto 3. Kuvaa vifaa vya kujikinga wakati wakugusa maji 4. Kuacha maji yaliyochotwa yatulie (Walau masaa 8) kabla ya kuyatumia 5. Kuchemsha maji au kuyaanika juani kabla ya kunywa 6. Nyingine (Fafanua)………………………………………………   88 Sijui |
| **TABIA ZINAZOHUSIANA NA KUGUSA MAJI** | | |
| 1. 23 | Kwa kawaida huwa unaenda ziwani? | 1. Ndiyo **→ Endelea swali namba 14** 2. Hapana**→ Endelea swali namba 18** |
| 1. 24 | Kwa kawaida unakwenda ziwani kufanya nini? | 1. Kuoga 2. Uvuvi 3. Kuchota maji 4. Kufua/kuosha vyombo 5. Kucheza/kujiburudisha 6. Kuogelea 7. Kusafiri/kwa kutumia maji ya ziwa mf. boti 8. Nyingine (Fafanua)…………………………………………………. |
| 1. 25 | Kwa kawaida huwa unaambatana na mzazi wako unapoenda ziwani? | 1. Ndiyo 2. Hapana |
| 1. 28 | Kwa wastani huwa wanatumia muda gani kukaa ziwani unapokwenda nao huko? | 1. Muda mfupi sana, < 5 dk 2. Muda mfupi, 5 - 15 dk 3. Muda mrefu, dk 15 – saa 1 4. Muda mrefu sana, > saa 1 na zaidi   88 Sijui |
| 1. 33 | Ni mda gani huwa unaenda/mnaenda ziwani? | 1. Asubuhi 2. Mchana 3. Jioni |
|  | Umewahi kushiriki / kupata dawa za minyoo shuleni | 1 Ndiyo  2 Hapana |
|  | Mara ngapi umepata dawa hizo |  |
|  | Kwa mara ya mwisho dawa hizo zilipatikana lini? |  |

**ELEZEA SEHEMU INAYOFUATA**

Sasa nitasoma sentensi zifuatazo na nitaomba unieleze kwa kiasi gani unakubaliana au unapingana na sentensi hizo. Baada ya kusoma kila sentensi tafadhali niambie endapo Unakataa kabisa, Unakataa, Hukubali wala hukatai, Unakubali au Unakubali kabisa

**D: Mtazamo juu ya ugonjwa wa kichocho**

| **No.** | **Questions and filters** | **Coding categories** |
| --- | --- | --- |
|  | Kichocho cha tumbo ni miongoni mwa magonjwa hatari na unaweza kusababisha kifo kwa watoto  **Unakataa kabisa, Unakataa, Hukubali wala hukatai, Unakubali, Unakubali kabisa?** | 1. Nakataa kabisa  2. Nakataa  3. Sikubali wala sikatai  4. Nakubali  5. Nakubali kabisa |
|  | Kuogelea/kuoga/kucheza katika maji ya ziwa inachangia uenezaji wa kichocho cha tumbo  **Unakataa kabisa, Unakataa, Hukubali wala hukatai, Unakubali, Unakubali kabisa?** | 1. Nakataa kabisa  2. Nakataa  3. Sikubali wala sikatai  4. Nakubali  5. Nakubali kabisa |
|  | Kujisaidia hovyo haja kubwa na kutokutumia choo vinachangia kwa kiasi kikubwa maambukizi mapya na kuenea kwa kichocho  **Unakataa kabisa, Unakataa, Hukubali wala hukatai, Unakubali, Unakubali kabisa?** | 1. Nakataa kabisa  2. Nakataa  3. Sikubali wala sikatai  4. Nakubali  5. Nakubali kabisa |
|  | Walezi wanachangia kwa kiasi kikubwa watoto wao kupata maambukizi ya kichocho kwa kuwapeleka katika vyanzo mbalimbali vya maji. Mf. Ziwa/mto/chemchem/ bwawa  **Unakataa kabisa, Unakataa, Hukubali wala hukatai, Unakubali, Unakubali kabisa?** | 1. Nakataa kabisa  2. Nakataa  3. Sikubali wala sikatai  4. Nakubali  5. Nakubali kabisa |
|  | Damu kwenye kinyesi ni mojaya dalili za kichocho cha tumbo  **Unakataa kabisa, Unakataa, Hukubali wala hukatai, Unakubali, Unakubali kabisa?** | 1. Nakataa kabisa  2. Nakataa  3. Sikubali wala sikatai  4. Nakubali  5. Nakubali kabisa |
|  | Maumivu ya tumbo ni moja ya dalili za kichocho cha tumbo  **Unakataa kabisa, Unakataa, Hukubali wala hukatai, Unakubali, Unakubali kabisa?** | 1. Nakataa kabisa  2. Nakataa  3. Sikubali wala sikatai  4. Nakubali  5. Nakubali kabisa |
|  | Inawezekana kujikinga dhidi ya Kichocho  **Unakataa kabisa, Unakataa, Hukubali wala hukatai, Unakubali, Unakubali kabisa?** | 1. Nakataa kabisa  2. Nakataa  3. Sikubali wala sikatai  4. Nakubali  5. Nakubali kabisa |
|  | Matumizi sahihi ya vyoo yanaweza kudhibiti Kichocho  **Unakataa kabisa, Unakataa, Hukubali wala hukatai, Unakubali, Unakubali kabisa?** | 1. Nakataa kabisa  2. Nakataa  3. Sikubali wala sikatai  4. Nakubali  5. Nakubali kabisa |
|  | Dawa za hospitali zinaweza kutibu/kuzuia kichocho | 1. Nakataa kabisa  2. Nakataa  3. Sikubali wala sikatai  4. Nakubali  5. Nakubali kabisa |
|  | Watoto wa shule za msingi wanatakiwa kupewa dawa za kutibu kichocho (Praziquantel). | 1. Nakataa kabisa  2. Nakataa  3. Sikubali wala sikatai  4. Nakubali  5. Nakubali kabisa |
|  | Dawa za kienyeji zinaweza kutibu/kuzuia kichocho | 1. Nakataa kabisa  2. Nakataa  3. Sikubali wala sikatai  4. Nakubali  5. Nakubali kabisa |
|  | Dawa za kutibu kichocho zinaweza kusababisha madhara makubwa ya kiafya hata kifo | 1. Nakataa kabisa  2. Nakataa  3. Sikubali wala sikatai  4. Nakubali  5. Nakubali kabisa |
