# Supplementary material for: Burden and risk factors for Schistosoma mansoni infection among primary school children: A quantitative school-based cross-sectional survey in Busega district, Northern Tanzania
Source: PLoS One. 2023 Jan 12;18(1):e0280180. doi: 10.1371/journal.pone.0280180 (PMC9836289; doi:10.1371/journal.pone.0280180)
Supplement: S2 File — (DOCX) [file pone.0280180.s002.docx]

# S2 File-English questionnaire

**Burden and risk factors for *Schistosoma mansoni* infection among Primary School Children: a quantitative school-based cross-sectional survey in Busega district, Northern Tanzania**

**Introduction**

Good morning/afternoon. My name is…………………………….. I am from the Muhimbili University. I am researching intestinal schistosomiasis among primary school-aged children in this community. I would like to learn and understand more from your experience on how intestinal schistosomiasis is transmitted. I am glad that you have accepted to talk to me. All information that will be gathered from you will be used without mentioning your names. This questionnaire interview will take about 20-30 minutes.

In case of any questions, please try to address them before we proceed.

May I now ask you a few questions related to your background information?

| **Questionnaire Identification ID** |  |
| --- | --- |
| Interviewer’s codes |  |

**A: Socio-demographic characteristics**

| **No.** | **Questions and filters** | **Coding categories** |
| --- | --- | --- |
|  | Sex of the respondent | 0 Female  1 Male |
|  | How old are you? (Age in complete years) |  |
|  | Which class level are you in? |  |

**B. Knowledge and Practices about schistosomiasis**

| **No** | **Questions and filters** | **Coding categories** |
| --- | --- | --- |
|  | Have you ever heard of a disease called schistosomiasis? | 1 Yes-continue with question 5  0 No-End the Survey |
|  | Where did you hear about it? (Circle as many responses as mentioned) | 1 School  2 Radio  3 Television  4 Health facility  5 Family/friends/neighbors  6 Health programs  7 Others, Specify………. |
|  | Mention the types of Schistosomiases that you know (circle as many responses as mentioned) | 1 Intestinal schistosomiasis (*S. mansoni*)  2 Urinary schistosomiases (*S. haematobium*)  3 Others  Specify………………  88 Don’t know |
|  | How do people get infected with schistosomiasis? (Circle as many responses as mentioned) | 1 Walking in the (contact with) infected lake/river water  2 Bathing in the lake/river  3 Swimming in the lake/river  4 Drinking dirty water  5 Eating contaminated food  6 Stepping onto faeces/urine of the infected person  7 Working out in the rain  8 Sexual contacts  9 Drinking unboiled water  10 Others, specify……………….  88 Don’t know |
|  | What symptoms does an infected person with intestinal schistosomiasis present with? (Circle as many responses as mentioned) | 1 Abdominal pain/colic  2 Diarrhea/loose stool  3 Blood in school  4 Bloody diarrhea (dysentery)  5 Vomiting blood  6 Swollen abdomen/ascites  7 Urinating drops of blood  8 Painful micturition  9 Swelling of the lower limb/another part of the body  10 Anemia  11 Flatulence/dyspepsia  12 Body weakness/fatigue  13 Others, specify………….  88 Don’t know |
|  | What behaviors contribute to the spread of intestinal schistosomiasis in children? | 1 Open defecation (e.g. bushes)  2 Swimming in water sources  3 Playing in dirty water  4 Playing with sand/soil  5 Eating dirty things  6 Walking barefooted  7 Others, specify…………………. |
|  | How does schistosomiasis get spread into the water bodies (lakes, rivers, ponds, etc.)? Circle as many responses as mentioned | 1 Urinating in the water body  2 Defaecating in the water body  3 Disposing of leftover foods in the water body  4 Witchcrafts  5 Sexual intercourses  6 Others, specify……………  88 Don’t know |
|  | Do you think that snails in the water bodies have anything to do with schistosomiasis? | 1 Yes  0 No  88 Don’t know |
|  | How do you avoid being infected with intestinal schistosomiasis? (Circle as many responses as mentioned) | 1 Taking preventive treatment e.g., MDA  2 Avoid swimming/bathing in water bodies e.g., lakes, river  3 Wearing protective clothing while in contact with water  4 Leaving fetched water to settle (for at most 8 hrs.) before using it  5 Boiling water or leaving it out in the sun before drinking it  6 Others, specify………………………………  88 Don’t know |
| **WATER CONTACT PRACTICES** | | |
|  | Do you go to the lake? | 1 Yes- continue with question 14  2 No- continue with question 18 |
|  | For what purpose do you go to the lake? | 1 Bathing  2 Fishing  3 Fetching water  4 Washing clothes/utensils  5 Playing/recreation  6 Swimming  7 Transport/fording in transit in lake water e.g., boats  8 Others, specify……………………… |
|  | Do you normally go with your parents to the lake? | 1 Yes,  2 No |
|  | On average, how long do you stay at the lake every time you go? | 1 Very short time, <5mins  2 Short time, 5-15minutes  3 Long time, 15 mins-1 hours  4 Very long time, >1 hour  88 Don’t know |
|  | At what time of the day? | 1 Morning  2 Midday  3 Afternoon |
|  | Have you ever participated in MDA? | 1 Yes  2 No |
|  | How many rounds did you participate in? |  |
|  | When was the last round of MDA? |  |

**EXPLAIN THE NEXT SECTION**

Now I am going to read some statements and I would like you to tell me how much you agree or disagree with them. After I read each statement, please tell me whether strongly disagree, disagree, neutral, agree, or strongly disagree.

**C: Attitudes towards schistosomiasis**

| No. | **Questions and filters** | **Coding categories** |
| --- | --- | --- |
|  | Intestinal schistosomiasis is a serious disease that can cause death among Children | 1. Strongly disagree 2. Disagree 3. Neutral 4. Agree 5. Strongly agree |
|  | Swimming/bathing/playing in the lake water facilitates the transmission of intestinal schistosomiasis | 1. Strongly disagree 2. Disagree 3. Neutral 4. Agree 5. Strongly agree |
|  | Open defecation and non-use of toilets contribute greatly to the re-occurrence and transmission of schistosomiasis | 1. Strongly disagree 2. Disagree 3. Neutral 4. Agree 5. Strongly agree |
|  | Parents/guardians greatly contribute to their children getting infected with intestinal schistosomiasis by taking them to various water sources e.g., lakes/rivers/streams/ponds | 1. Strongly disagree 2. Disagree 3. Neutral 4. Agree 5. Strongly agree |
|  | Blood in the stool is one of the symptoms of intestinal schistosomiasis | 1. Strongly disagree 2. Disagree 3. Neutral 4. Agree 5. Strongly agree |
|  | Stomach/abdominal pain is one of the symptoms of intestinal schistosomiasis | 1. Strongly disagree 2. Disagree 3. Neutral 4. Agree 5. Strongly agree |
|  | Schistosomiasis can be prevented | 1. Strongly disagree 2. Disagree 3. Neutral 4. Agree 5. Strongly agree |
|  | The proper use of the latrine can control schistosomiasis | 1. Strongly disagree 2. Disagree 3. Neutral 4. Agree 5. Strongly agree |
|  | Modern medicine cures/prevents schistosomiasis | 1. Strongly disagree 2. Disagree 3. Neutral 4. Agree 5. Strongly agree |
|  | Primary school children should be given praziquantel, the drug that is given to school-aged children to treat and prevent schistosomiasis | 1 Strongly disagree  2 Disagree  3 Neutral  4 Agree  5 Strongly agree |
|  | Traditional medicine cures/prevents schistosomiasis | 1 Strongly disagree  2 Disagree  3 Neutral  4 Agree  5Strongly agree |
|  | Drugs used to treat and prevent schistosomiasis can cause serious effects or death | 1 Strongly disagree  2 Disagree  3 Neutral  4 Agree  5 Strongly agree |
