# Supplementary material for: Burden and risk factors for Schistosoma mansoni infection among primary school children: A quantitative school-based cross-sectional survey in Busega district, Northern Tanzania
Source: PLoS One. 2023 Jan 12;18(1):e0280180. doi: 10.1371/journal.pone.0280180 (PMC9836289; doi:10.1371/journal.pone.0280180)
Supplement: S3 File — (DOCX) [file pone.0280180.s003.docx]

**S3 File. Observation checklist**

***Schistosoma mansoni* field sanitary inspection form**

**A General information**

| **Field /Household location and information**  (Record information on household location and population. Add “N/A” where information is not applicable.) | | | | | |
| --- | --- | --- | --- | --- | --- |
| **House name/no.** | **Village/town** | **Community** | | **District** | **Province** |
|  |  |  | |  |  |
| **Additional location information:**  (If using coordinates, state the type and unit e.g. national grid reference coordinates; GPS coordinates.) | | |  | | |

***B. Schistosoma mansoni* sanitary inspection activities**

| **Sanitary inspection variables** | | **NO**  (Tick) | **YES**  (Tick) | **COMMENTS** |
| --- | --- | --- | --- | --- |
| 1 | A habit of people defecating close to the lake |  |  |  |
| 2 | A habit of people bathing in lake water source |  |  |  |
| 3 | A habit of children playing/swimming in the lake |  |  |  |
| 4 | People farming close to the lakeshores |  |  |  |
| 5 | People fishing in the lake |  |  |  |
| 6 | People collecting water for domestic use from the lake |  |  |  |
| 7 | A habit of people washing bicycles in lake water |  |  |  |
| 8 | Presence of toilets close to the lake where people carry out their daily activities the lake |  |  |  |
| 9 | Presence of vegetation cover/algal mass for snail habitat |  |  |  |
| 10 | Stagnant water body which favors snail habitat |  |  |  |

Designation/title of Inspector: ...............................................................

Signature: ............................... Date: .....................................................
