# Supplementary material for: Burden and risk factors for Schistosoma mansoni infection among primary school children: A quantitative school-based cross-sectional survey in Busega district, Northern Tanzania
Source: PLoS One. 2023 Jan 12;18(1):e0280180. doi: 10.1371/journal.pone.0280180 (PMC9836289; doi:10.1371/journal.pone.0280180)
Supplement: S4 File — (DOCX) [file pone.0280180.s004.docx]

**S4 File. Snail collection and report form**

Snail collection and report **form per sampling site**

| **Snail ID** |  |
| --- | --- |
| **Site of collection** |  |
| **Cercarial shedding status** |  |
| **Date of shedding**  **(dd/mm/yy)** |  |
| **Snail species**  **Collection method** |  |
| **Time of shedding**  **(morning/evening)** |  |
| **Snail preservation method** |  |
| **Comments** |  |
| **Examined by**  **Name:**  **Designation:** |  |
| **Verified by**  **Name:**  **Designation:** |  |
